# Date Palm (*Phoenix dactylifera*) Fruits as a Potential Cardioprotective Agent: The Role of Circulating Progenitor Cells

**DOI:** 10.3389/fphar.2017.00592

**Published:** 2017-09-04

**Authors:** Ibrahim A. Alhaider, Maged E. Mohamed, K. K. M. Ahmed, Arun H. S. Kumar

**Affiliations:** ^1^Department of Pharmaceutical Sciences, College of Clinical Pharmacy, King Faisal University Al-Ahsa, Saudi Arabia; ^2^Department of Pharmacognosy, Faculty of Pharmacy, Zagazig University Zagazig, Egypt; ^3^Phcog.Net Bengaluru, India; ^4^Stemcology, University College Dublin Dublin, Ireland

**Keywords:** antioxidant, CD34^+^, CD133^+^, flavonoid, phenolic compounds

## Abstract

**Context:** Date palms, along with their fruits’ dietary consumption, possess enormous medicinal and pharmacological activities manifested in their usage in a variety of ailments in the various traditional systems of medicine. In recent years, the identification of progenitor cells in the adult organ systems has opened an altogether new approach to therapeutics, due to the ability of these cells to repair the damaged cells/tissues. Hence, the concept of developing therapeutics, which can mobilize endogenous progenitor cells, following tissue injury, to enhance tissue repair process is clinically relevant.

**Objectives:** The present study investigates the potential of date of palm fruit extracts in repairing tissue injury following myocardial infarction (MI) potentially by mobilizing circulating progenitor cells.

**Methods:** Extracts of four different varieties of date palm fruits common in Saudi Arabia eastern provision were scrutinized for their total flavonoid, total phenolic, *in vitro* antioxidant capacity, as well as their effects on two different rodent MI models.

**Results:** High concentrations of phenolic and flavonoid compounds were observed in date palm fruit extracts, which contributed to the promising antioxidant activities of these extracts and the observed high protective effect against various induced *in vivo* MI. The extracts showed ability to build up reserves and to mobilize circulating progenitor cells from bone marrow and peripheral circulation to the site of myocardial infraction.

**Conclusion:** Date palm fruit extracts have the potential to mobilize endogenous circulating progenitor cells, which can promote tissue repair following ischemic injury.

## Introduction

Despite tremendous growth in pharmacotherapy of myocardial infarctions (MIs), mortality continues to rise (Caplice and Doyle, 2005; Saleem et al., 2008; Nofal and Abdulmohsen, 2010). Permanent loss of cardiomyocytes after ischemic injury results in irreversible loss of myocardial contractility, reduction in ventricular performance, and may initiate the development of dilated heart failure (Nofal and Abdulmohsen, 2010). The discovery that pluripotent progenitor cells (stem cells) bearing the capacity to differentiate into mature cardiac cells raised the hope of cell-based regenerative medicine (Saleem et al., 2008). Grafting of stem cells in the damaged myocardium, repair, and functional improvement appeared suddenly a nearby reality (Kumar et al., 2009). Recently, there was a tremendous focus on learning the role of vascular progenitor cells in the treatment of several cardiac and vascular disorders (Dawn et al., 2005). Several adult and embryonic precursor cell populations have reported to differentiate the vascular cell phenotype and participate in a range of biological repair functions within the cardiovascular system. The cell therapy era as moved from the preclinical arena to the clinical phase, wherein potential benefits are widely reported with the use of progenitor cells in the context of cardiovascular disorders, especially MI. However, considerable preclinical work is needed to further refine the progenitor cell therapy-based medicine (Kawamoto and Asahara, 2007; Jujo et al., 2008; Yamahara and Itoh, 2009; Adamo and Garcia-Cardena, 2012; Brunt et al., 2012).

*Phoenix dactylifera*, commonly known in Arabic as Nakl and in English as date palm, is a well-known monocot plant and a member of the Arecaceae family. Date palm is a major fruit crop in Saudi Arabia where the approximate of 24 million trees produce just about a million ton of dates per annum accounting for 15% of world’s production. As in other countries of the Gulf and in the Middle East, date palm is closely associated to the life of people with a high socioeconomic importance due to not only its food value, but also its valuable supply of vitamins, mineral fibers, and carbohydrates (Al-Farsi et al., 2005a; Baloch et al., 2006; El Hadrami and Al-Khayri, 2012). Worldwide, 2000 or more date cultivars are known to exist (Ali-Mohamed and Khamis, 2004). Saudi Arabia has a rich diversity of about 400 date cultivars of which 10 varieties including Khalas, Sheshi, and Reziz are popular and have a high consumer preference, especially in the Eastern province of the Kingdom (Anonymous, 2006). Various parts of date palm are applied in folk medicine as a remedy for various illnesses such as fever, inflammation, nervous disorders, loss of consciousness, and dementia. Several medicinal properties have been reported for the date palm such as antiatherogenic and antioxidant activity (Chaira et al., 2009), antiviral activity (Jassim and Naji, 2010), gastroprotective effects (Al-Qarawi et al., 2003, 2005), antimutagenic properties (Vayalil, 2002), and hepatoprotective effects (Saafi et al., 2011). There are plenty of published manuscripts describing other medicinal and pharmacological activities of date palm fruits such as its anti-inflammatory effect (Boghdadi et al., 2012; Metwaly et al., 2012), immuno-modulatory activity (Karasawa et al., 2011), antimicrobial effect (Boulenouar et al., 2011), neuroprotective activity in diabetic peripheral neuropathy (Zangiabadi et al., 2011), and anticancer activity (Elberry et al., 2011).

Even with extensive studies on the medicinal and pharmacological properties of dates and its constituents, the effects of date fruit consumption on heart and cardiovascular system are not well-investigated yet although the proved activity in traditional health systems and folk medicine. Most of cardiovascular-related effect of date palm relay on the fruit effect on cholesterol and lipid profile as well as the fruit well-distinguished effect as an antioxidant. Only one review discussed the direct effect of date palm fruits on the heart muscle in cases of cardiomyopathy (Al-Yahya et al., 2016). In this report, the authors indicated that “Ajwa,” a variety of date palm fruits, consumption by rats prevented the depletion of endogenous antioxidants such as superoxide dismutase (SOD) and catalase (CAT) and inhibited lipid peroxidation. Ajwa downregulated the expressions of proinflammatory cytokines (IL-6, IL-10, and TNFα) and apoptotic markers and upregulated the anti-apototic protein Bcl2 indicating antioxidant, hypolipidemic, anti-inflammatory, and anti-apoptotic potential against myocardial damage.

The present study explores the date palm fruits’ therapeutic effects in improving cardiovascular defects in MI. The study mainly aims to investigate as possible and potential activity of these fruits as mobilizers of endothelial progenitor cells (CD133^+^ or CD34^+^) and hence explaining their beneficial effects in the therapy of cardiovascular diseases.

## Materials and Methods

### Collection and Extraction of Plant Materials

Four different varieties of date palm (*Phoenix dactylifera*) fruits (Berhi (D1), Khalase (D2), Khenizi (D3), and Reziz (D4)) were identified and collected from the local markets or farms in Ahsaa (eastern) region of Saudi Arabia. All the date samples were collected in the edible stage and identified by experts and taxonomists in the date and palm center, King Faisal University, Ahsaa, Saudi Arabia with specimens deposited to the herbarium of the same center. A total of 250 g of the different four varieties were macerated in 75% ethanol in distilled water using cold maceration techniques (Vazirian et al., 2012) for 48 h, filtered, and then the alcohol was evaporated under reduced pressure and the remaining aqueous extracts were freeze-dried. Stock solution (1 mg/ml) of different extract was prepared in methanol (D1–D4).

### Determination of Total Flavonoid and Total Phenolic Contents

The total flavonoidal and phenolic contents of various extracts have been determined according to Olajuyigbe and Afolayan (2011). Folin–Ciocalteu reagent was used in the assay of total phenolic content using gallic acid as standard. The concentration of phenolic contents was described as gallic acid equivalents (GAEs) for 1 g of extract.

For flavonoid content determination, methanolic aluminum chloride (2% v/v) was used and absorbance of the yellow color developed was measured at 420 nm. Quercetin was used as a standard and concentration of flavonoids was expressed as mg Quercetin equivalent (QE) for 1 g of the tested extract.

### Antioxidant Activity

Total antioxidants capacities of different extracts were determined *in vitro* using 2,2-diphenyl-1-picrylhydrazyl (DPPH; Brand-Williams et al., 1995; Mathiesen et al., 1995), reducing power assay (RPA; Benzie and Szeto, 1999; Koleva et al., 2002), Hydroxyl radical scavenging assay (HRSA; Nehete et al., 2010), and superoxide anion radical scavenging activity (SARSA; Chen and Yen, 2007; Gülçin et al., 2010). Ascorbic acid was used as a standard in DPPH, HRSA, and SARSA while Quercetin was use as a standard in RPA.

### Toxicity Studies

All animal experimental procedures and protocols were approved by Animal Research Ethics Committee at King Faisal University, Saudi Arabia and performed in accordance with the Guidelines for Ethical Conduct for Use of Animals in Research, King Faisal University (unless otherwise specified). Male Westar rats (200 ± 10 g, 6–8 weeks), used in animal experiments, were obtained from the Animal Breeding Center, King Saud University, Riyadh, Saudi Arabia (unless otherwise specified). Animals were housed with food and water and maintained in standard laboratory conditions, at temperature 25 ± 1°C and relative humidity 55 ± 5% with a 12 h light/dark cycle. Acute toxicity and lethality of the extracts were determined by using the method described by Lorke (1983). Rats were orally administered with 50, 100, 1000, 1500, 3000, and 5000 mg/kg of the each extract, respectively, and observed for 24 h for death. There was no mortality or toxic signs were recorded in any of the groups treated with date palm fruits extracts.

### Anti-myocardial Infarction Activity

Different extracts were investigated for its anti-myocardial infarction activity using the *in vivo* isoproterenol model (Ahmed et al., 2004; Prabhu et al., 2006; Bhandari et al., 2008). The rats were randomly divided into 10 groups with six rats each (*n* = 6). Group 1, normal animals, received saline 10 ml/kg body weight with standard food and water to allow *ad libitum* throughout the experimental period. Group 2, rats were orally fed normal saline once daily for 28 days and in addition received isoproterenol (85 mg/kg body weight) on the 29th and 30th day at an interval of 24 h. Groups 3–10, rats were pretreated with dates extract (D1–D4) (200 and 400 mg/kg body weight, respectively) for a period of 28 days and in addition received isoproterenol 85 mg/kg body weight) on the 29 and 30 day at an interval of 24 h. All the animals from respective groups were observed for heart weight, body weight changes, and mortality. At the end of the treatment period, rats were anesthetized with pentobarbitone sodium (60 mg/kg) and serum was separated from the blood for the estimation of cardiac biomarkers: troponin-T, lactate dehydrogenase (LDH), creatinine kinase (CK), and aspartate transaminase (AST) using respective kits according to the manufacturer protocols. Heart was excised immediately and immersed in physiological saline. It was suspended in 10% (w/v) ice-cold 0.1 M phosphate buffer (pH 7.4) and cut into small pieces. The required amount was weighed and homogenized using a Teflon homogenizer (Inco, India). The clear supernatant was used for estimation of thiobarbituric acid reactive substance (TBARS), reduced glutathione (GSH), SOD, and CAT using respective kits according to the manufacturer protocols.

### Histopathological Examination

The histopathological studies were performed as described (Ahmed et al., 2004; Prabhu et al., 2006; Bhandari et al., 2008). The hearts were isolated and washed immediately with ice cold saline then fixed in 10% formalin. After fixation tissues were embedded in paraffin-wax and thick sections were cut into thin sections and stained with hematoxylin and eosin. These slides were then observed under light microscope (Leica DM4, at power 100×) for histopathological changes.

### Effect on Circulating CD34 and CD133 Positive Progenitor Cells

C57BL6J mice (males, ∼25 g) were used for this study with 14 mice in each group. Mice were purchased from Charles River, United Kingdom, and maintained on a standard commercial rodent diet. After acclimatization to the facility, mice were treated with the date palm fruit extracts (D1, D2, D3, and D4) at 100, 200, or 400 mg/kg/day orally for 28 days. On day 15 post plant extracts treatment, myocardial infarct–reperfusion was surgically administered in the mice as below and were followed for further 14 days.

Myocardial infarction: The study was approved and performed in accordance with the guidelines of the Institutional Animal Research Ethics Committee of University College Dublin, Dublin (Approval number AE18982/I166), Ireland. Myocardial infarction was induced in male C57BL6/J mice by temporary ligation of left anterior descending (LAD) artery for 40 min followed by continuous reperfusion. Mice were anesthetized using a combination of urethane (1.25 g/kg), ketamine (90 mg/kg), and xylazine (10 mg/kg). The animals were intubated and ventilated with a Rodent ventilator (mouse vent^®^, Kent Scientific Corporation, United States). Body temperature was monitored and maintained at 37°C during the procedure. Following left thoracotomy between the 3rd and 4th intercostal space, LAD artery was ligated using 8-0 suture over a PE tubing placed 0.5 mm below the tip of the left atrium. Myocardial infarction was visually confirmed by observation of left ventricular pallor immediately post ligation. Forty minutes following MI, the suture was cut and removed to facilitate continuous reperfusion. Thoracotomy was closed and the animal was maintained under mechanical ventilation until spontaneous breathing occurred and recovered in a thermos-regulated chamber set at 37°C. A mortality rate of 8.5% was observed in the first 48 h post-surgery. Fifty microliters of blood was sampled from facial vein at baseline (before surgery), 2 h, 24 h, 3rd, 7th, and 14th day post-surgery for analysis of CD34 and CD133 cells levels in peripheral blood. Additionally on 14th day, the mice were administered by over dose of xylazine ketamine anesthesia and the femurs were collected to isolate bone marrow sample to detect levels of CD34 and CD133 cells in bone marrow.

Flow cytometry: Cells from peripheral blood, and bone marrow were collected for cytometric analysis using a BD Accuri^TM^ C6 cytometer (Becton Dickinson, Belgium). Peripheral blood and bone marrow mononuclear cells (MNCs) were isolated by densitometric separation and stained with antibodies against CD 34 (Anti-CD 34 PE conjugated, Catalog number 50589-R013-A-100, Sino Biological Inc.) and CD 133 (Anti-CD133 DyLight 650 conjugated, Catalog number NB120-16518C, NOVUS Biological Inc.). Each sample was run in duplicate. Reported percentages were gated on MNC singlet population. Appropriate isotype controls (rabbit IgG), as well as unstained samples, were used as negative controls.

### Statistical Analysis

Values are expressed as mean ± SEM and analyzed using Graph Pad prism version 5.1 using two-way ANOVA followed by Tukey’s multiple comparison test (*post hoc* test). *P* < 0.05 was considered significant.

## Results

### Determination of Total Flavonoid and Total Phenolic Contents

**FIGURE 1:**
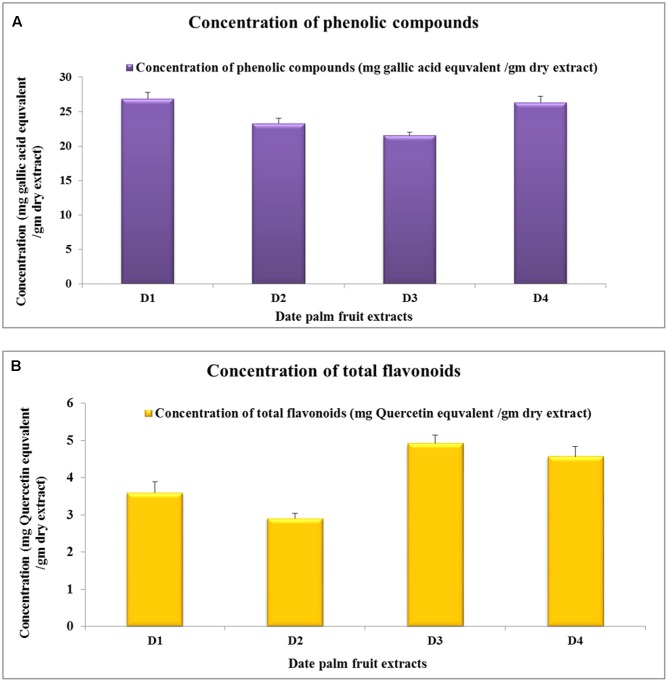
Phenolic compounds and flavonoids concentrations in date palm fruit extracts. **(A)** Concentration of total phenolic components in different extract (calculated as mg of gallic acid equivalents/grams of extract). **(B)** Concentration of flavonoid components in different extract (calculated as mg of Quercetin equivalent/grams of extract). The values expressed as mean of six independent samples (*n* = 6) ± SEM.

The determination of total flavonoid and phenolic contents showed that the amount of these components has some differences among the tested extracts of the date palm fruit. The quantity of total phenolic contents varied from 21.53 ± 0.90 to 26.82 ± 0.92 mg GAE/g of dry plant material (**Figure 1A**). The flavonoid contents ranged from 2.90 ± 0.13 to 4.92 ± 0.21 mg QE/g of dry plant material (**Figure 1B**). Generally, D1 extract contains the highest percentages of total phenolic content, followed by D4 extract, then D2 leaving D3 with the least amount of these compounds. On contrary, D3 extract contains the highest percentages of total flavonoid contents, followed by D4 extract, then D1 extract leaving D2 extract with the least amounts of these components.

### Antioxidant Activity

Total antioxidants capacities of deferent extracts were determined *in vitro* applying four methods: DPPH, RAP, HRSA, and SARSA. In all assays except RAP, 50% inhibition concentration (IC_50_) was calculated as a way to assess different extracts’ antioxidant properties in comparison with a known standard, usually ascorbic acid.

**FIGURE 2:**
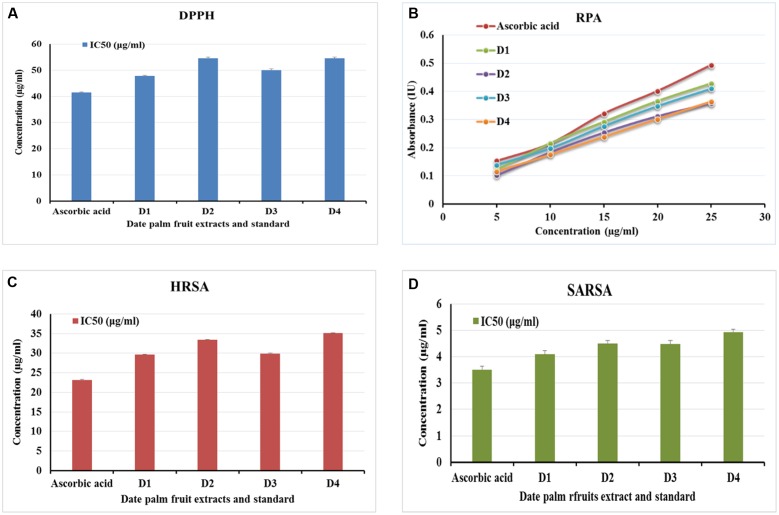
*In vitro* antioxidant effect of date palm (*Phoenix dactylifera*) fruits. Extracts of date palm fruits varieties [Berhi (D1), Khalase (D2), Khenizi (D3), and Reziz (D4)] were subjected to four *in vitro* antioxidant assays: 2,2-diphenyl-1-picrylhydrazyl (DPPH; **A**), reducing antioxidant power assay (RAP; **B**), hydroxyl radical scavenging activity (HRSA; **C**), and superoxide anion radical scavenging activity (SARSA; **D**). The values expressed as mean of six independent experiments (*n* = 6) ± SEM.

The results showed that high DPPH scavenging activity of all extracts ranged from IC_50_ of 47.82 ± 0.267 μg/ml to 54.58 ± 0.524 μg/ml. Comparing this activity with the standard reveals that the antioxidant power of all extracts was not significantly different from that of ascorbic acid (**Figure 2A**).

Similar results can be seen in the RPA. The highest reducing activity was observed by D1 followed by D3 extracts (**Figure 2B**); however, no significant difference can be seen among all extracts’ activities or when compared to the standard, ascorbic acid.

Results for both HRSA and SARSA showed high scavenging activity of all extracts ranged from IC_50_ of 29.62 ± 0.0967 μg/ml to 35.13 ± 0.108 μg/ml in HRSA and IC_50_ of 4.09 ± 0.134 μg/ml to 4.93 ± 0.111 μg/ml in SARSA. Comparing those activities with the standard reveals that the antioxidant power of all extracts was not significantly different from that of ascorbic acid (**Figures 2C,D**).

### Anti-myocardial Infarction Activity

**FIGURE 3:**
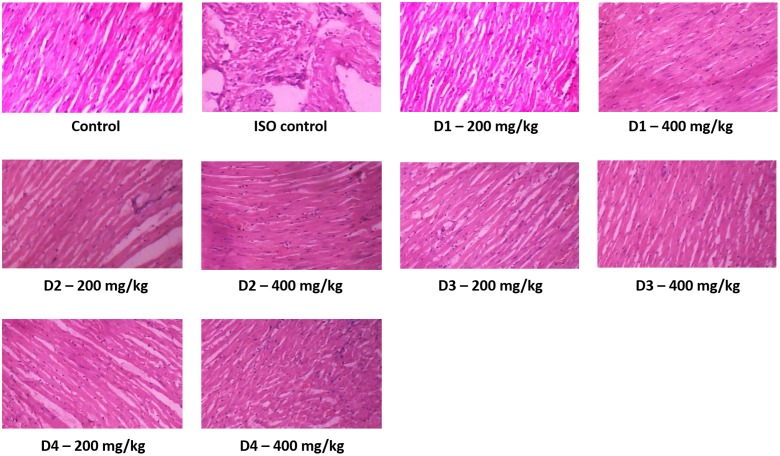
The cardiac tissue histopathological photos after induction of myocardial infarction (100×). *Control* rat animals received saline 10 ml/kg with standard fed and water *ad libitum* throughout the experimental period. *ISO control* rats were orally fed normal saline once daily for 28 days and in addition received isoproterenol (85 mg/kg body weight) on the 29th and 30th day at an interval of 24 h. Date extract rats (*D1–D4*) were pretreated with dates extract (D1–D4) (200 and 400 mg/kg body weight, respectively) for a period of 28 days and in addition received isoproterenol 85 mg/kg body weight) on the 29th and 30th day at an interval of 24 h. These slides were then observed under light microscope for histopathological changes.

The examination of the anti-myocardial infarction activity of extracts was performed to check whether these extracts possess anti-myocardial infarction activity as well as to assess the ability of these extracts to restore the serum and cardiac enzymes in isoproterenol-induced MI in rats to or near to the normal state. The results showed that isoproterenol hydrochloride (ISO) has already induced the state of MI as appeared in the levels of serum and tissue biomarkers (**Table 1**) as well as from the histopathological examination (**Figure 3**). Pretreated with dates extract (D1–D4) for a period of 28 days prior to isoproterenol injection significantly improved the state of MI and this can be suggested from the levels of tissue and serum biomarker, which shows significant difference from the ISO control group (**Table 1**). Meanwhile, many of the biomarkers levels are approaching the normal control levels again indicating high activity of date palm fruit extract against the induced MI. The dose change from 200 to 400 mg/kg made significant difference in most cases the higher dose was favored. Administration of date palm fruits from different samples elevated the levels of GSH, SOD, and CAT, and diminished the levels of TBRS in the heart tissues indicating the protective effect of these extracts (**Table 1**). In the same time, the consumption of date palm fruit extracts nearly restores the normal values of troponin-T, LDH, CK, and AST in serum. D2 extracts (200 mg/kg dose) ameliorated the levels of GSH, CAT, and CK by 200, 237, and 42% (calculated from **Table 1**), respectively, while the 400 mg/kg dose enhanced the levels of CAT and LDH by 207 and 54%, respectively. D4 extracts (200 mg/kg dose) intensified the GSH, CK, and AST by 187, 38, and 61%, respectively, while reduced the TBARS levels by 62% (calculated from **Table 1**). The 400 mg/kg dose of D4 extracts enhanced the GSH and AST by 206 and 57%, respectively, and decreased troponin-T levels by 78.5% (calculated from **Table 1**).

**Table 1:** Effect of date palm fruit extracts on myocardial tissue antioxidant parameters [thiobarbituric acid reactive substance (TBARS), reduced glutathione (GSH), superoxide dismutase (SOD), and catalase (CAT)] and on serum marker enzymes [troponin-T, lactate dehydrogenase (LDH), creatinine kinase (CK), and aspartate transaminase (AST)].

Treatment groups	TBARS (nmol/g wet wt)	GSH (μg/g wet wt)	SOD (IU/mg protein)	CAT (IU/mg protein)	LDH (IU/l)	CK (IU/l)	AST (IU/l)	Troponin-T (μg/ml)
Control	8.3 ± 0.15	172.8 ± 8.8	1.6 ± 0.32	52 ± 3.2	134.0 ± 2.4	104.0 ± 0.81	128.0 ± 2.0	0.6 ± 0.12
ISO control	67.4 ± 7.00^a^	85.33 ± 2.5^a^	0.29 ± 0.01^b^	27 ± 4.4^b^	283.0 ± 3.2^a^	236.0 ± 2.8^a^	326.0 ± 2.0^a^	2.8 ± 0.32^a^
D1 – 200 mg/kg	20.4 ± 1.9^∗∗∗^	144.2 ± 13.5^∗∗^	1.9 ± 0.28^∗∗∗^	59 ± 2.4^∗∗∗^	162.0 ± 3.6^∗∗∗^	181.0 ± 1.2^∗∗∗^	235.0 ± 1.6^∗∗∗^	0.7 ± 0.04^∗∗∗^
D1 – 400 mg/kg	27.9 ± 4.2^∗∗∗^	208.3 ± 15.1^∗∗∗^	1.8 ± 0.20^∗∗∗^	63 ± 1.6^∗∗∗^	132.0 ± 1.2^∗∗∗^	151.0 ± 0.81^∗∗∗^	220.0 ± 3.6^∗∗∗^	0.8 ± 0.08^∗∗∗^
D2 – 200 mg/kg	15.8 ± 1.0^∗∗∗^	257.5 ± 7.6^∗∗∗^	2.2 ± 0.28^∗∗∗^	91 ± 3.2^∗∗∗^	195.0 ± 1.6^∗∗∗^	136.0 ± 2.0^∗∗∗^	231.0 ± 4.8^∗∗∗^	0.5 ± 0.04^∗∗∗^
D2 – 400 mg/kg	22 ± 2.6^∗∗∗^	268.2 ± 11.7^∗∗∗^	3.7 ± 0.12^∗∗∗^	83 ± 2.4^∗∗∗^	128.0 ± 2.8^∗∗∗^	196.0 ± 3.2^∗∗∗^	218.0 ± 0.81^∗∗∗^	0.9 ± 0.12^∗∗∗^
D3 – 200 mg/kg	22.7 ± 3.6^∗∗∗^	251.2 ± 7.9^∗∗∗^	2.7 ± 0.36^∗∗∗^	86 ± 2.0^∗∗∗^	189.0 ± 2.4^∗∗∗^	143.0 ± 2.8^∗∗∗^	195.0 ± 3.2^∗∗∗^	0.6 ± 0.04^∗∗∗^
D3 – 400 mg/kg	23.9 ± 4.5^∗∗∗^	264.7 ± 10.0^∗∗∗^	1.8 ± 0.04^∗∗∗^	102 ± 4.8^∗∗∗^	131.0 ± 1.6^∗∗∗^	123.0 ± 2.8^∗∗∗^	186.0 ± 1.2^∗∗∗^	0.7 ± 0.04^∗∗∗^
D4 – 200 mg/kg	25.3 ± 3.9^∗∗∗^	245.7 ± 13.0^∗∗∗^	2.9 ± 0.08^∗∗∗^	96 ± 1.4^∗∗∗^	151.0 ± 2.8^∗∗∗^	146.0 ± 0.81^∗∗∗^	126.0 ± 2.8^∗∗∗^	0.5 ± 0.08^∗∗∗^
D4 – 400 mg/kg	17.1 ± 1.7^∗∗∗^	261.8 ± 8.6^∗∗∗^	2.9 ± 0.12^∗∗∗^	79 ± 1.2^∗∗∗^	162.0 ± 1.2^∗∗∗^	119.0 ± 2.0^∗∗∗^	140.0 ± 2.0^∗∗∗^	0.6 ± 0.04^∗∗∗^


*All values expressed as mean ± SEM of six independent samples (n = 6)*. ^a^*p < 0.0001 vs. control*. ^b^*p < 0.001 vs. control*. ^∗∗^*p < 0.001 vs. ISO control*. ^∗∗∗^*p < 0.0001 vs. ISO control*.

### Histopathological Examination

Histopathological analysis showed normal cardiac muscle fibers with cellular integrity in normal untreated rats. In ISO group, the histopathological inspection showed that isoproterenol induced area of infarction characterized by split cardiac muscle fibers, edematous intramuscular spaces, and inflammatory infiltrates. Isoproterenol also caused lesions disorders of muscle fibers and myocardial structure as well as permeation of red blood cells (**Figure 3**) with disturbance of the cardiomyocytes architecture. Treatment with date palm fruits extract moderated isoproterenol-induced changes in cardiac tissues and improved the tissue lesions in dose-dependent manner (**Figure 3**). The pretreatment date palm fruits extract reduced necrosis, edema, and restored the cardiomyocytes architecture and preserved cardiac muscle fiber morphology. The 400 mg/kg doses gave better cardiomyocytes architecture preservation with less lesion and vacuoles than the 200 mg/kg doses. No differences can be seen in histopathology of cardiac muscles between the four varieties of date palm fruits.

### Effect on Circulating CD34 and CD133 Positive Progenitor Cells

**FIGURE 4:**
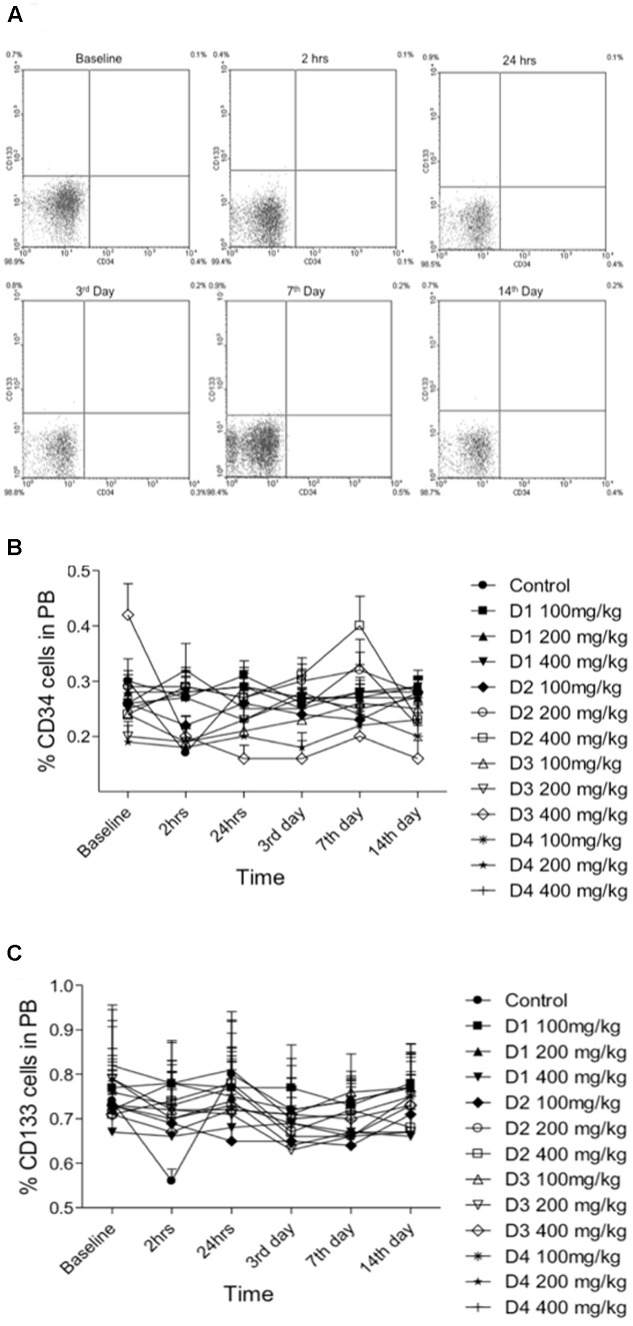
Levels of CD34 and CD133 positive cells in peripheral blood at baseline (before induction), 2 h, 24 h, 3rd, 7th, and 14th day post-myocardial infarction (MI) induction. **(A)** Representative FACS data of peripheral blood. **(B,C)** Percentage number of CD34 and CD133 positive cells, respectively, for each concentration (100, 200, and 400 mg/kg) of date palm fruit extracts (D1, D2, D3, and D4).

**FIGURE 5:**
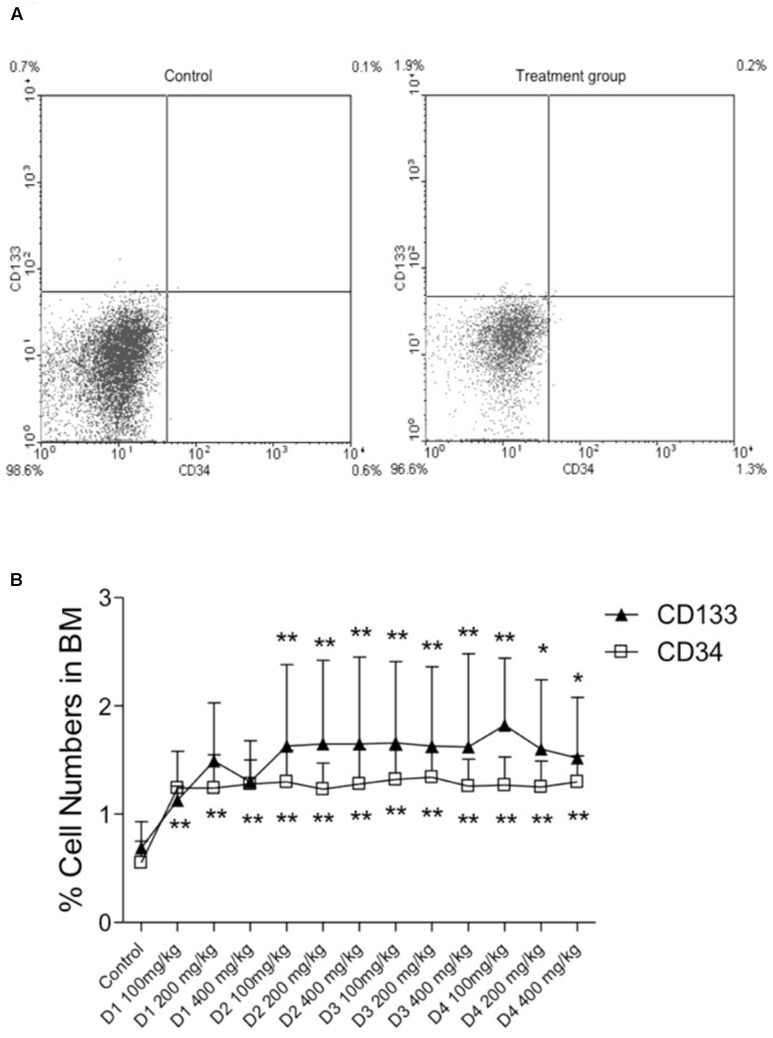
Levels of CD34 and CD133 positive cells in bone marrow at 14 days post-MI. **(A)** Representative FACS data of bone marrow. **(B)** Percentage number of CD34 and CD133 positive cells for each concentration (100, 200, and 400 mg/kg) of date palm fruit extracts (D1, D2, D3, and D4). Symbols indicating the significant differences: ^∗^*p* < 0.01 vs. control, ^∗∗^*p* < 0.001 vs. control.

Two hours post-myocardial infarction (MI) we observed a drop in the circulating levels of the CD34 and CD133 positive cells, which was returned to normal levels by 24 h post-MI and these levels were sustained up to 14 days post-MI (**Figure 4**). Interestingly, the drop in circulating levels of the CD34 and CD133 positive cells at 2 h post-MI was significantly prevented in groups pretreated with the date palm extracts (D1, D2, and D4) for 28 days (**Figure 4**). However, this effect of extract D3 was observed only for CD133 but not CD34 positive cells (**Figures 4B,C**). We presumed that the effects of date palm extract to prevent fall in the circulating levels of CD34 and/or CD133 positive cells post-MI may be due to the ability of these extracts to build the reserves of these progenitor cells in the bone marrow. Indeed, when we assessed the levels of CD34 and CD133 positive cells in the bone marrow at 14 days post-MI, we observed the levels of these cells to be significantly higher in date palm extract-treated groups compared to the control group (**Figure 5**).

## Discussion

Palm date fruits are known for their high food values and their medicinal uses in the Arab world. These fruits have been used for centuries to the extent that it becomes involved in the peoples’ tradition and socio-economics. The study, hereby, spot the light on the date palm fruit useful effects on the cardiovascular system especially in the case of MI which is a consequent of angina and ischemic heart diseases.

The date palm fruit extracts contain various concentrations of phenolic and flavonoid compounds, to which the noteworthy antioxidant activity of these extracts could be attributed. The best antioxidants compounds known are the phenolic compounds, particularly flavonoids (Al-Farsi et al., 2005b; Chaira et al., 2009; Olajuyigbe and Afolayan, 2011). Various hypotheses are suggested for the beneficial effects of these compounds in improving the poor health associated with environmental pollution-induced lung and micro vascular diseases and compromise immune system (Nijveldt et al., 2001). Flavonoids have long been recognized to possess anti-inflammatory, antioxidant, antiallergic, hepatoprotective, antithrombic, and antiviral properties (Folts, 2002; Al-Farsi et al., 2005b; Vayalil, 2012) as they show scavenging activities for free radicals through acting as electron-donating and/or metal ion chelating agents (Rice-Evans and Burdon, 1993). Phenolic compounds and flavonoids have also been recognized for their effect on MI especially on the outcomes of experimentally induced disease by isoproterenol or other models (Kavitha et al., 2015; Khalil et al., 2015).

Pretreatment with dates extract for 28 days prior to isoproterenol injection improved the state of MI, implied from the levels of tissue and serum biomarker and histopathological investigation. Date extracts normalized many biomarkers levels indicating high activity of these extracts against the induced MI. Histopathological investigation of normal myocardial tissue showed that cellular integrity in normal untreated rats without any evidence of edema and infiltration of neutrophils. The isoproterenol-treated rats show extensive myofibrillar degeneration, infiltration of inflammatory mediators like neutrophils along with coagulative necrosis. Treatment with date palm fruits extract moderated isoproterenol-induced changes in cardiac tissues and improved the tissue lesions in dose-dependent manner. The pretreatment date palm fruits extract reduced necrosis, edema, and restored the cardiomyocytes architecture and preserved cardiac muscle fiber morphology. The 400 mg/kg dose gave better cardiomyocytes architecture preservation with less lesion and vacuoles than the 200 mg/kg dose. Isoproterenol-induced necrotic damage related to increased level of lipid peroxidation. The palm fruits extract treatment significantly decreased the elevated lipid peroxidation indicating that inhibition of isoproterenol induced necrotic damage along with neutrophil infiltration in the myocardium.

Circulating bone marrow cells offer an effective means to repair tissue injury especially when the biochemical conditions are favorable toward such repair functions. This may probably be the reason why we observed a drop in the circulating levels of CD34 and CD133 positive cells at 2 h post-MI, wherein the progenitor cells are mobilized from peripheral circulation to the site of tissue injury (MI in this case). Therapeutic or preventive measures, which can build the reserves of these circulating progenitor cells, will be beneficial as this ensures the ready availability of the progenitor cells for tissue repair both structurally and possibly by paracrine mechanisms. Indeed, we observed an increase in the reserves of both CD34 and CD133 positive cells in the bone marrow following treatment with date palm extracts (D1, D2, D3, and D4) for 28 days. This build up the CD34 and CD133 positive cells reserve in the bone marrow could most likely have avoided the drop in the circulating levels of these cells at 2 h post-MI in the treatment group. Hence, such preventive and therapeutic approaches may prove to be valuable adjunct to current therapeutic approaches in medical management of patients post-MI, which warrants further studies. A few recently published studies show the potential beneficial effects of flavonoids on endothelial progenitor cells (Kumar et al., 2009); however, it is likely that such beneficial effects may also be on other bone marrow-derived progenitor cells, which may have several health benefits. For instance, bone marrow-derived progenitor cells was enhanced in chronic smokers consuming green tea (Kumar et al., 2009), and such effects may be attributed to the antioxidant nature of flavonoids in the green tea (Hertog et al., 1993). Thus, it is likely that flavonoid constituents of the natural products contribute to the beneficial effects on progenitor cells.

## Conclusion

In a summary, the state of MI in mouse model was improved by consumption of date palm extracts from different varieties and different concentrations. Khalase (D2) and Reziz (D4) samples were the most active date palm varieties and the 400 mg/kg dose was more favored. All date varieties tested exhibited high concentrations of phenolic and flavonoid components as well as high antioxidant activities. All the date palm varieties built up the CD34 and CD133 positive cells reserve in the bone marrow and mobilized these progenitor cells toward the site of myocardial injury resulting in tissue repair and improvement of the state of MI. The discovery of such effects to date palm fruits has increased its medicinal values and enriched the diversity of its pharmacological activities, therefore this study recommend the consumption of date palm fruits among patients with history or risk of developing ischemic heart disease.

## Author Contributions

This study was conceptualized and designed by IA, KA, and AK. KA prepared the samples and performed toxicity studies. KA and MM performed the phenolic and flavonoid content experiments. IA and MM performed the antioxidant experiments. IA and KA performed the MI model with isoproterenol, measured serum and tissue parameters, and made the histopathological study. AK made the MI model 2 by temporary ligation of LAD artery and measured all the parameters. All authors interpreted the data, discussed the results, and contributed to preparing the manuscript.

## Conflict of Interest Statement

The authors declare that the research was conducted in the absence of any commercial or financial relationships that could be construed as a potential conflict of interest.
